# Assessment of nurses knowledge and skills following cardiopulmonary resuscitation training at Mbarara Regional Referral Hospital, Uganda

**DOI:** 10.11604/pamj.2018.30.108.15398

**Published:** 2018-06-11

**Authors:** John Bosco Tamu Munezero, Catherine Atuhaire, Sara Groves, Samuel Nambile Cumber

**Affiliations:** 1 School of Medicine, Department of Nursing Sciences, Kabale University, Uganda; 2 Faculty of Medicine, Department of Nursing, Mbarara University of Science and Technology, Uganda; 3 Faculty of Medicine, Department of Nursing, John Hopkins University, USA; 4 Section for Epidemiology and Social Medicine, Department of Public Health, Institute of Medicine, The Sahlgrenska Academy at University of Gothenburg, Box 414, SE-405 Gothenburg, Sweden

**Keywords:** Cardiopulmonary resuscitation, knowledge, nurse, training, manikin, Uganda

## Abstract

**Introduction:**

Cardiopulmonary resuscitation (CPR) is considered a core emergency skill in which all health care professionals must be proficient. CPR remains a new procedure in developing compared to develop countries. The objective of this study was to assess Nurses Knowledge and Skills following Cardiopulmonary Resuscitation Training at Mbarara Regional Referral Hospital.

**Methods:**

A prospective pre/post intervention design was adopted. CPR knowledge and skills of 32 nurses from MRRH were assed using two tools. **Tool I** consisted of 17 item of multiple choice questions that assessed CPR knowledge. **Tool II** involved an observation checklist of 15-point skills questions. A penalty score of 5 or 10 or 20 was set for each question, based on the guideline.

**Results:**

The average score prior to instruction was 53.8 for knowledge and posttest 82.5, and for skills was 46 pre-instruction and 81.5 post instruction. There was a statistically significant (p < 0.001) improvement in the CPR knowledge and (p = 0.02) for CPR skills. The percentage change in respondent's knowledge and skills ranged from 16.8% to137.2% with a mean of 59.9% for knowledge and from 19.18% to 2115.6% with a mean of 159.8% for the skills assessment.

**Conclusion:**

Respondents had inadequate CPR knowledge and skills at pretest. The study revealed statistically significant improvement in both knowledge and skills of CPR for all nurses post training. There was a significant change in nurses' skills than in knowledge post training.

## Introduction

Cardiopulmonary Resuscitation (CPR) is a critical component of Basic Life Support (BLS) and Advanced Life Support (ALS) [1]. CPR is an important lifesaving procedure that can keep cardiac arrest patient alive long enough for definitive treatment to be delivered [1]. Literature indicates deficiencies in Nurses CPR knowledge and skills [2]. In western world and other developed countries, nurses CPR knowledge and skills also differs [3,4]. CPR knowledge and skills depend on how frequent trainings were given [5]. Compulsory training is required every 2 years for employment in all licensed health facilities in the USA [6]. It appears, there is no established CPR training schedules for hospital-based healthcare providers In Uganda and other developing countries like Kenya and Nepal [2,7]. This indicates an existing CPR education and practice gap. Cardiopulmonary resuscitation (CPR) is the combination of chest compressions and rescue breathing. It forms the basis of modern BLS [8]. Effective CPR is vital for survival of cardiac arrest victims. The chances of survival in cardiac arrest decreases by between 7 and 10% for each minute of CPR delay [8]. It could also decrease the number of days prior to hospital discharge [9]. Cardiac arrest is the cessation of normal circulation of blood due to failure of the heart to contract effectively [10]. Various internationally and nationally accepted guidelines for CPR have been published, and formal training programs based on these guidelines have been conducted by certified training centers [11]. Uganda appears not to be having any certificated body of CPR that is responsible for CPR training and certification. Clinical and CPR procedure guidelines also appear to be limited in Ugandan Health Facilities. Nurses employed in critical care units used to be trained in CPR by anaesthiologist and lacked continuous supervision and evaluation. This has compounded the CPR education and practice for the nurses. Quality CPR is required to save life of cardiac arrest victim. It is significant for health professions to successfully perform such lifesaving skills that they rarely perform. This makes it imperative for regular retraining in CPR [12]. It cannot be overemphasized that CPR is a critical procedure in cardiac arrest that may be rarely performed but has to be competently done when it is necessary. In Uganda, literature about CPR training and subsequent changes in knowledge and skills among medical professionals more especially nurses appears limited. In Uganda, nurses are more likely to identify cardiac arrest both in the emergency/critical care units and medical/surgical wards than any other health professional and clinical and CPR procedure guidelines appears to be limited. This affects the nurse's potential to initiate CPR. Nurses rarely initiated and performed CPR on cardiac arrest Victims at Mbarara hospital. This could be due to a gap in their education and practice. No studies were found that reported assessment of knowledge and skills of CPR and regular training of nurses. This creates a need to assess nurses' knowledge and skills following CPR training at Mbarara regional referral hospital in western Uganda. This study was therefore designed to assess Nurses knowledge and skills following CPR training among all nurses working in the emergency/critical care units and medical/surgical wards given that there are more likely to identify cardiac arrest patients. This is likely to highlight the need for regular in service CPR training programs to keep nurses updated and competent in CPR.

**Broad objectives:** The study assessed nurse's knowledge and skills following CPR training at Mbarara regional referral hospital in western Uganda.

**Researcher hypothesis:** Nurses' CPR knowledge and skills will change after the training and there will be differences in mean/median scores between the pretest scores and posttest scores.

**Specific objectives:** 1) assessment of nurses' knowledge and skills of CPR pre training at Mbarara Regional Referral Hospital in western Uganda; 2) assessment of nurses' knowledge and skills of CPR post training at Mbarara Regional Referral Hospital in western Uganda; 3) assessment of change in knowledge and skills of CPR among nurses after training at Mbarara Regional Referral Hospital in western Uganda.

## Methods

In this study:

**Knowledge of CPR** means ability of a person to have correct understanding of CPR in terms of definition, causes, signs & symptoms, complications, and actions to prevent sudden death from cardiac arrest.

**Skills of CPR means** Routine activities and actions of individual or group to prevent cardiac arrest, improve the Patient's chance of survival and increase the opportunity of recovery.

**Study Setting:** Mbarara Regional Referral Hospital (MRRH) in western Uganda is approximately 265 kilometers (165 miles), southwest of Kampala in Mbarara District in the Ankole sub-region, and is located within Mbarara Municipality. It is the referral hospital for the districts of Mbarara, Bushenyi, Buhweju, Mitooma, Rubirizi, Sheema, Ntungamo, Kiruhura, Ibanda and Isingiro. The hospital serves as the teaching hospital for Mbarara University of Science and Technology. Its bed capacity is 350. The study adopted a prospective pre/post intervention design that utilized quantitative methods.

**Population:** The study was carried out among all cadres of qualified nurses working with adults in the Accidents and Emergency units (A&E), Intensive Care Unit (ICU), operating theatres (OT) and medical and surgical wards at MRRH. All qualified nurses on duty in above units and wards. Nurses who were taking care of critically ill patients.

**Sample Size** [13]: Yamane (1967) provides a simplified formula to calculate sample size. This formula was used to calculate the sample sizes A 95% confidence level and P = 0.5 are assumed for the equation below.

$$\text{n}=\frac{\text{N}}{1+\text{N}{\left(\text{e}\right)}^{2}}$$

N= total number of nurses from units that were considered estimated to 47 nurses (hospital's personnel office October 2015). n = desired sample size e = desired level of precision at 5%, with a 95% confidence interval when the above equation is applied to the sample of nurses used,

$$\text{n}=0\text{.5}\left[1-0\text{.5}\right]/\left[\left({\left(0\text{.05}\right)}^{2}/{\left(1\text{.96}\right)}^{2}\right)+\left(0\text{.5}\left[0\text{.5}\right]/45\right)\right]\text{/0.95}=42$$

**Sampling technique:** The study targeted 42 nurses working in MRRH. Non-probability convenience sampling was utilized and only 32 participants from considered units participated in the study. Convenience sampling was appropriate since the participants simply happen to be in a convenient place at a convenient time [14]. During the pre-test, training and post-test, all the 32 nurses participated.

**Data collection tools:** The data was collected using two tools; tool I: this consisted of 17 multiple choice questions that assessed nurses' knowledge about cardiac arrest, and CPR (definition, causes, signs &symptoms, complications, and actions to prevent sudden death). The tool was revised by 2 supervisors A pass mark of 85%, as recommended by the [15] and according to *Basic Life Support for health care provider's* document of the Resuscitation Council of Southern Africa (RCSA) [16] exists for CPR knowledge test for certification was used since no standardized guidelines appeared to exist in the hospital and in Uganda that indicated the pass mark.

**Tool II:** This involved a 15-point skills test that was adjusted to assess CPR skills in a limited resource context of Ugandan nurses. The steps that related to use of defibrillation were dropped due limited availability of defibrillators. According to [17], planning CPR training effectively, requires assessment of the available resources and the environment in which the training will take place. This skills test used the same approach similar to that of [18] and [5] that was also used to assess the nurses' CPR skills. A maximum penalty mark of 5 or 10 or 20 was set for each step based on that approach and AHA guideline. The researcher assessed each participant´s performance during the pre-test and the posttest immediately after the CPR training. The validity of the content was enhanced by following the current guidelines of CPR specified by the [15]. The tools were also revised by 2 supervisors.

**Data collection procedure:** During the pre-test phase each nurse completed demographic characteristics and multiple-choice questions assessing his/her CPR knowledge. A 4 hour session was arranged with permission of hospital administration and the charge nurses. A written and skills pre-test was completed by each nurse after a demographic survey and tool I assessment. Nurse's CPR skills were assessed for 5 minutes on CPR mankin. 3-hour CPR training session based on the AHA 2015 guidelines was given. CPR training manikin designed especially for BLS training was used to simulate the features of an average adult physiology and anatomy. CPR knowledge (written test) and skills of the participants were re-assessed immediately after the training.

**Ethical considerations :** Once departmental approved was obtained, an introductory letter from Head of the Nursing Department was attached on submission to FRC and then to Research Ethics Committee (REC). The ethical approval for the study was obtained from the REC of Mbarara University of Science and Technology. We obtained approval and permission to conduct the study from the Director of MRRH. Participation was voluntary and informed written consent was provided by each participant. The right to decline or to withdraw from the study at any stage without incurring any penalty was explained. All data were coded and kept locked up, accessible only to the researcher. There were no risks or adverse effects encountered. The content validity of the data was enhanced by following the guidelines of CPR specified by the AHA. The tools were also pre-tested on five nurses 2 of which were registered nurses, 2 certificate nurses and 1 graduate nurse who did not participate in the actual study. During the tool evaluation and pre-testing phase the five participant's encountered difficulties in understanding of two of the 19 questions and one of the questions lacked updated information and were dropped. The 15 steps in skills assessment were graded and penalty points assigned, based on the participants' levels of performance'. Penalty point was a score which was deducted from a participant whenever he /she performed a task wrongly. This made instrument easy to score and awarded penalty points in a similar way during the pre-test phase and posttest phase.

**Data analysis:** The data were sorted, coded and entered into the Statistical Package for Social Sciences (SPSS) version 20. Data were analysed using the SPSS Version 20. Descriptive statistics (Frequencies, proportions, mean and standard deviation) were obtained. Test of normality was done and the sample yielded a non- normal distribution. This warranted use of non-parametric tests that are distribution free statistical tests. A number of non-parametric tests (Friedman Test, Mann-Whitney U Test, Wilcoxon signed Rank Test and Kruskal-Wallis Test) were used. Friedman test was used in this study to test whether or not the difference in the CPR knowledge and skills before training and after training was statistically significant. The Mann-Whitney U test is used to analyze ordinal data with 95% of the power test (t-test) to detect differences between 2 independent groups [14]. In this study it was used to analyze the mean scores of the pretest, post-test and significant differences of CPR knowledge and skills in work experience groups. The Wilcoxon matched-pairs signed-ranks test is a non-parametric test used to examine the changes that occurred in the pre-test-post-test measures or in the matched-pairs measures [14]. This test was used in this research to assess the change in the pre-test and post-test a mean scores. P value of < 0.05 was considered to be statistically significant.

**Limitations of the study:** The research was confined to one-referral hospital in Western Uganda, which limits the inferences about other populations of nurses in Uganda. The non-probability sampling methods used limits generalizability to only the study group in study areas. The study was conducted using mannikins that may not provide the participants with the same competence as a real-life situation would have provided. Participants were therefor encouraged to practice more amongst themselves and rotate on patients requiring CPR.

## Results

**Respondents' demographic characteristics.** Table 1: out of the 32 respondents, 65.6% (n = 21) were females and 34.4% (n = 11) were males. Most respondents were between 26 and 48 years old with a mean age of 34.81 years. They had 2 to 24 years of experience with a mean of 10.66 years of experience. Based on education, 62.5 % (n=20) were certificate level nurses, 31.3% (n=10) were at the diploma level and 6.3% (n=2) were bachelors level nurses. As many as 56.3% (n= 18) of the respondents had no formal CPR training, 37.5% (n = 12) had received prior CPR training during their service as nurses and 6.2 % (n=2) reported having had prior BLS training. One respondent, 3.1% performed CPR on a weekly basis, whilst 40.6% (n = 13) did so monthly, 40.6% (n=13) did so once a year and 15.3% (n=5) had never performed CPR.

**Table 1:** Respondents’ sociodemographic characteristics

		Frequency (N=32)	Percentage (%)
**Gender**	Male	11	34.4
	Female	21	65.6
**Qualification**	Certificate	20	62.5
	Diploma	10	31.3
	Bachelors	2	6.3
**Experience category**	5 years or less	7	21.9
	6-10 years	11	34.4
	11-15 years	8	25.0
	16-20 years	5	15.6
	21 years & above	1	3.1
**Working area**	ICU	6	18.8
	A&E	8	25
	Theatre	6	18.8
	Medical	6	18.8
	Surgical	6	18.8
**Prior training in CPR**	BLS certificate	2	6.2
	ACLS certificate	0	0.0
	In service CPR training	12	37.5
	No prior training	18	56.3
**Previous CPR performance**	Daily	0	0.0
	Often (every week)	1	3.1
	Not often (once in a month)	13	40.6
	Once a year	13	40.6
	Never	5	15.3

**Respondents pretest and posttest knowledge and skills of CPR:** The table below shows distribution of scores in the four groups and the Friedman test that assed for the significance of distribution of scores within each group. Sample yielded a non- normal distribution and Friedman test was an appropriate non parametric test for more than 2 related samples. Table 2: the pretest scores knowledge and skills ranged from 33.3% to 72.2% and 3.2% and 77, 4% respectively. The average pre-test score on the knowledge was 53.8% and 46.0% for skills. Following the instructional intervention, the post test score ranged from 72.0 to 95.2% for knowledge and 70.9% and 95% for skills. The post-test mean score improved to 82.5% knowledge and 81.5% skills. The Friedman test revealed a significant difference in scores in four related samples X^2^ (3) = 79.35, p < 0.001. Post hoc pairwise comparisons did not indicated difference between pretest skills score and pretest knowledge scores.

**Table 2:** Respondents pretest and posttest knowledge and skills of CPR

Friedman Test for related samples						
	N	Mean	Std. Deviation	Minimum	Maximum	Mean rank
**pre-test knowledge score**	32	53.816	11.3895	33.3	72.2	1.75
**pre-test skills score**	32	46.000	17.0193	3.2	77.4	1.25
**post-test knowledge score**	32	82.453	6.5217	72.0	95.2	3.56
**post-test skills score**	32	81.534	6.7901	70.9	95.0	3.44
						

Test statistic: X2 (3) = 79.35, p<0.001

**Pretest and post-test scores of respondents' knowledge and skills and qualification level:** Table 3 indicates the mean and standard deviation of CPR scores of knowledge and skills in 3 category of work experience in accordance with professional qualification. Due to a non-normal distribution, Kruskal-Wallis which is an equivalent of one Analysis of Variance (ANOVA) was used to test the significance in relationship between qualification and pretest and posttest scores of CPR knowledge and skills (Table 3). The respondents' scores increased after the training in all the 3 professional qualification levels. The mean scores increased with the level of education both in the pretest and posttest. a Kruskal-Wallis test, a non-parametric test revealed no significant effect of qualification levels in either the pretest or post test score of knowledge.

**Table 3:** Professional qualification and scores of knowledge and skills at pre-test and post test

Kruskal-Wallis Test					
		CPR knowledge		CPR skills	
Professional qualification		Pretest	Posttest	Pretest	Posttest
**Certificate**	Mean	52.730	79.69	41.76	79.70
	Std. Deviation	11.58	6.3	17.48	6.31
**Diploma**	Mean	54.3	82.950	54.83	85.32
	Std. Deviation	11.60	6.28	13.53	5.77
**Bachelor**	Mean	62.25	83.00	44.20	81.00
	Std. Deviation	7.03	4.24	19.51	12.72
**Test statistic**		X2(2)= 1.476, p = 0.478	X2 (2)= 0.77, p = 0.680	X2(2) = 4.416, P =0.110	X2(2) = 5.905, p = 0.52

**Pre-test and post-test scores of respondents' knowledge and skills and their work experience:** Table 4 shows the distribution of respondent's scores of CPR knowledge and skills in 2 categories of work experience. It also shows the relationship between work experience and pre-test and posttest scores of CPR knowledge and skills. A Mann-Whitney Test which is an equivalent of 2 independent samples t-test was used to test the significant differences of CPR knowledge and skills scores in the two independent samples according to work experience (Table 4).

**Table 4:** Work experience and scores of knowledge and skill at pre-test and posttest

Mann-Whitney Test								
	Work experience	N	Mean	Minimum	Maximum	Std. Deviation	Mean Rank	Test statistic (p value)
**pre-test knowledge score**	10 years and below	18	49.43	33.3	62.1	11.32	12.94	*U* = 62.0*, p=* 0.01
	Above 10 years	14	59.46	42.1	72.2	8.99	21.07	
	Total	32						
**post-test knowledge score**	10 years and below	18	80.93	72.2	94.4	5.88	14.17	*U* = 84.0*, p = 0.15*
	Above 10 years	14	84.41	72	95.2	6.99	19.50	
	Total	32						
**pre-test skills score**	10 years and below	18	38.26	3.2	69.0	15.83	12.22	*U*= 49.0, p = 0.003
	Above 10 years	14	58.95	37	77.4	13.17	22.00	
	Total	32						
**post-test skills score**	10 years and below	18	79.44	70.9	93.5	6.57	13.25	*U* = 67.5*, p* = 0.0*3*
	Above 10 years	14	84.22	74.4	95.0	6.29	20.68	
	Total	32						

**Knowledge.** Mean pre and posttest scores of respondents with over 10 years work experience were higher at 59.42% and 84.06% respectively compared to those with less than 10 years of experience whose mean scores pre and post intervention were 49.43% and 80.93%. Difference in the scores between the experience levels were significant at the pretest assessment, *U = 62.0 ,p= 0.014*, but following the educational intervention there was no statistical difference, *U = 84.0 , p = 0.116* in the knowledge between nurses with less than 10 years of experience and those with greater than 10 years of experience.

**Skills.** Respondents with work experience of less than 10 years had mean scores of 38.26% and 79.44% in pretest and posttest skills respectively, whilst those with work experience of above 10 years had mean scores of 58.95% and 84.22% in pretest and posttest skills respectively. A Mann- Whitney test revealed a statistically significant difference in the skills scores between the experience levels at the pretest assessment, *U = 49.0, p = 0.003* and following the educational intervention there was a statistical difference in skills scores at posttest, *U = 67.5, p=0.026* in the knowledge between nurses with less than 10 years of experience and those with greater than 10 years of experience.

**Prior CPR training and scores of knowledge and skills of CPR at pre-test and posttest.** **Table 5**: The mean pretest knowledge scores were high in respondents who had prior CPR training compared to those who did not have prior CPR training. The mean scores for respondents with no prior CPR training, prior in-service CPR training and prior BLs training were 48.53%, 59.60% and 66.65% respectively. A Kruskal-Wallis test revealed that there was a significant effect of Prior CPR training on the pretest knowledge scores, X^2^(2) = 9.16, p = 0.01. Inspection of post hoc pairwise comparisons indicated differences between no prior CPR training category and prior in-service CPR training and having prior CPR training increased the scores. The mean posttest knowledge scores were high in respondents who had prior CPR training compared to those who did not have prior CPR training. The mean scores for respondents with no prior CPR training, prior in-service CPR training and prior BLs training were 80.99%, 82.36%, and 92.00% respectively. Respondents who had prior CPR training had still had high scores on posttest. There was no statistically significant effect of prior CPR training on the posttest scores, *X^2^(2) = 3.66, p=0.160.* The respondents' mean pretest skills scores within categories of no prior CPR training, prior in-service CPR training and prior BLS training were 40.55%, 50.02% and 70.95% respectively. Respondents who had prior CPR training had high scores than ones who did not have prior CPR training though the difference of pretest skills scores within the categories of prior CPR training was not statistically significant, X^2^(2) = 5.823, p = 0.054. The respondents' mean posttest skills scores within categories of no prior CPR training, prior in-service CPR training and prior BLS training were 80.14%, 82.37% and 89.00% respectively. Respondents who had prior CPR training had higher posttest skills scores than those who did not have prior CPR training however the differences in the posttest skills scores within the categories of prior CPR training was not statistically significant, *X^2^(2) = 3.87, P =0.144.*

**Table 5:** Prior CPR training and scores of knowledge and skills of CPR at pre-test and posttest

Scores					
Prior CPR training		pre-test knowledge score	post-test knowledge score	pre-test skills score	post-test skills score
**Prior BLS Certificate**	Mean	66.650	92.000	70.950	89.000
	Std Deviation	7.8489	4.2426	9.1217	7.0711
**Prior In Service Training**	Mean	59.600	80.992	50.017	82.375
	Std Deviation	7.1727	3.1742	12.6622	5.8820
**No prior Training**	Mean	48.533	82.367	40.550	80.144
		Std Deviation	11.2737	7.5988	17.4209	7.0458

**Respondents' gain scores and percentage change in knowledge and skills after training.** Table 6 shows the minimum, maximum, and mean points gained and percentage change in the CPR knowledge and skills. It also shows the non-parametric tests used to test statistical differences in the CPR knowledge and skills gain and percentage change. Table 6: among all the 32 respondents there was gain in both knowledge and skills. The gain in knowledge ranged from 11.9 points to 49.9 points and from 14.0 points to 74.1 points for skills. The mean knowledge gain was 28.6 points and 35.5 points for skills. Friedman test revealed a statistically significance differences in gain scores both in knowledge and skills of cardiopulmonary resuscitation, X^2^(2) = 6.125, P = 0.013. The knowledge percentage in respondents ranged from 16.8%-137.2% and 19.18% to 2115.6% for skills. The mean percentage change was 59.9 % for knowledge and 159.8% for skills. Respondents' skills improved considerably more than their knowledge. A Wilcoxon signed ranks test revealed a significant differences in the median between knowledge percentage change and skills percentage change, Z= -3.104, p = 0.002.

**Table 6:** Gain scores and percentage change in respondents’ knowledge and skills after training

Friedman test					
Gain in scores	N	Minimum	Maximum	Mean	Std. Deviation
Knowledge gain in scores	32	11.9	49.9	28.64	9.92
Skills gain in scores	32	14.0	74.1	35.53	14.65
**Test statistic**					**X^2^(2) = 6.125, P=0.013**
**Wilcoxon Signed Ranks Test**					
Percentage change	N				
Percentage change knowledge	32	16.88	137.24	59.9022	35.18203
Percentage change skills	32	19.18	2115.63	159.8489	368.45102

Test statistics for percentage change Z= -3.104, p = 0.002

Change= [100* posttest-pretest]/Pretest

**Hypothesis Testing.**

**Null hypothesis (H_0_):** Nurses' CPR knowledge and skills will not change after the training and the difference in the mean /median scores between the pretest and post test scores is zero.

**Researcher hypothesis (H_A_):** Nurses' CPR knowledge and skills will change after the training and there will be differences in mean/median scores between the pretest scores and posttest scores. The null hypothesis is rejected at 0.05, the median of diffences between percentage change in knowledge and skills is not 0. Nurses' CPR knowledge and skills improved after the training and there were differences in mean/median scores between the pretest scores and posttest scores.

## Discussion

**Outcome of pretest of CPR knowledge and skills prior to intervention.** There were differences in the pretest scores of respondents when categorized by working experience. Respondents with more experience had higher scores of knowledge and skills (Table 4), mean score were high with high qualification on pretest and not on posttest (Table 2 and Table 4), and mean scores were high among respondents with prior training in CPR (Table 5). There was a statistically significant effect of work experience on both knowledge and skills pretest scores. This concurs with findings of a study conducted in Belgium that indicated statistical significant association of accumulated work experience and improved CPR skills [19]. There was a statistically significant difference in knowledge at pretest scores within the categories of prior CPR training and not in pretest scores of CPR skills. There was no statistically significant effect of qualification levels on scores of either knowledge or skills at pretest and posttest. This however, could be because of a limited ability of this study to detect a significant difference given that the means were different. A larger sample may have provided a more normal distribution and more power to use a parametric ANOVA that could detect statistical differences. It has become apparent that competency in CPR skills and knowledge are fundamental to effective CPR interventions to save patients' lives. This study indicated that nurses in MRRH had low competences in CPR knowledge and skills at pretest assessment. This reduces nurse's initiation and performance of CPR. The average pre-test score of the respondents were 53.8% for knowledge and 46% for skills indicated that they had very low knowledge. These scores would be very low if compared to the [15,16] standard of 85%. Similar findings in a study done in Bahrain by [20] were reported where no nurse passed the CPR skills test based on the standard pass mark of 85. This study did not indicate enough evidence in previous training and practice levels of their study participants to use the standard pass mark. In this study given that majority (56.3%) had no prior CPR training and many (40.6% perform CPR once a year, the pretest mean score of 53.8% for knowledge and 46% for skills indicated that nurses were enthusiastic in practice and training. Most of the respondents prior to the instruction did not know when to assess the pulse, when to commence chest compressions, the correct compression/ventilation ratio, or the recommended compression rate. This finding concurs with a study that reported most of the personnel had weak competence in CPR skill. Differences of competence levels ranged from 0% to 100% and the CPR skill scores in performing cardiac compression was low [21]. These aspects are critical for initiating and performing effective CPR. According to [22] incorrect performance of these aspects of CPR reduces the chances of survival after a cardiac arrest. In addition, the low pretest knowledge and skills scores could be due to the fact that some participants had worked for over 10 years after their studies and could have forgotten over the years. The AHA guidelines also changes every 5 years, with CAB replacing ABC, changes in compression rates, when to do pulse check, compression depth, and compression to ventilation ratio have changed since 2000 or 2005.

**Outcome of posttest of CPR knowledge and skills post intervention.** The CPR knowledge and skills of the nurses did improve considerably post intervention given that there was no significant previous training and CPR performance from a pretest average scores of 46.0% to 81.3% for skills and 53.8% to 82.45% for knowledge. In several studies, the increase of nurses' knowledge after training was highlighted too [5, 23]. These skills are also likely to deteriorate with time as has been reported by studies done in Belgium [24] and in Ireland [5]. This suggests the need for continues practice and regular training to maintain and acquire competency. Regular training through in-service CPR training is therefore needed to attain and maintain CPR competency to increase the survival rate of cardiac arrest victims. This concurs with [25] who noted that teaching of the relevant, frequently used CPR knowledge and skills could increase the survival rate of cardiac arrest victims.

**Exploration of change in knowledge and skills of CPR after retraining.** There was a considerable and statistically significant gain (p = 0.013) in knowledge and in skills immediately after training intervention. Participants gained more points in skills than in knowledge post intervention (*mean of 159.8% increase for skills compared to a mean of 59.9% increase in the knowledge)*. More improvement in the respondents' skills of CPR than knowledge immediately after training was noted. This more improvement in skills could have been due to study implementation that stressed more on skill of Performing CPR as pre assessment had indicted low scores in skills compared to knowledge. In addition it could possibly have been due to the fact that the skill of performing a particular procedure like CPR is more grasped than knowledge during the practice and it is more likely to deteriorate with time if it not continuously practiced compared to knowledge. The median change differences in knowledge and skills were statistically significant (p = 0.02). The null hypothesis of no change in the nurses' knowledge and skills of CPR after training was rejected at 0.05. As for the magnitude of the improvement in knowledge and skills of respondents regarding the respondents age, working experience , area of work, prior CPR training and CPR performance , this study did not find any significant association. This could either be due to successful effect of study training to all nurses irrespective of their demographic characteristics or the fact that the study was under powered due to sample size to detect significant changes between the categories of the nurses by experience, age, area of work or level of qualification. The finding does not concur with those of a study that found out that younger nurses are more motivated in acquiring technical skills and knowledge [26]. It did not also concur with findings in a study done in South Africa that indicate, better knowledge and skills acquisition among staff working in accident and emergency departments than staff working in other areas of the hospital [27].

## Conclusion

Nurses in MRRH had inadequate CPR knowledge and skills at pre assessment, which could have negatively impacted the effective CPR performance. The study revealed statistically significant improvement in both knowledge and skills of CPR for all nurses post training. Effective CPR regular training needs to be instituted in MRRH to ensure continuous training and practice for the nurses to acquire competency and maintain the knowledge and skills. The training had considerable change in the nurses' knowledge and skills, more change noticed in skills than knowledge.

**Recommandation**: A similar study with triangulated design where concealed observation is included to determine the actual competences of nurses, pre and post training. A study to assess the Nurses' perceptions and barriers to CPR Knowledge and skills. A longitudinal follow study to determine critical points at which CPR knowledge and skills deteriorate. Experimental study with a larger sample size to determine the effectiveness of CPR training on Nurses CPR knowledge and skills.

**What is known about this topic**

- There were no established CPR training schedules for hospital-based healthcare providers Deficiencies in CPR knowledge and skills due to lack of retraining;
- Nurses rarely initiated and performed CPR on cardiac arrest Victims most likely due education and practice gap;
- No studies were found that reported assessment of knowledge and skills of CPR and periodic retraining of nurses.

**What this study adds**

- Respondents had inadequate CPR knowledge and skills which could have been due: a) deterioration since educational completion;b) lack of up to date CPR knowledge since the tests and skills check lists were based on the newest AHA 2015 guidelines that is updated every 5 years; c) lack of sustainable continuous in-service CPR training;
- The study indicated improvement in both knowledge and skills of CPR irrespective of age, experience, working area and level of qualification: **implication to practice:** a) inadequate knowledge and skills of CPR reduces nurse's initiation and performance of effective CPR hence potentially negatively affecting survival rates during cardiac arrest situations; b) unless effective CPR training is instituted and sustained, it is more likely that the death from cardiac emergencies is likely to continue to increase since quality CPR is fundamental in survival of cardiac emergence victims; c) improved knowledge and skills of cardiopulmonary resuscitation to detect and prevent cardiac arrest that is likely to improve critical clinical nursing care and practice;
- **Implication to policy:** a) need to institute regular refresher in-service CPR training in the study hospital for all nurses to improve, update and sustain nurses' CPR competency to provide evidence based nursing practice in CPR; b) nurse in charges need to arrange more continuous professional development in CPR based on current evidence to acquire skills and improve confidence and practice among nurses to initiate and perform CPR.

## Competing interests

The author declare no competing interests.
